# Autonomy versus support: self-reliance and help-seeking for mental health problems in young people

**DOI:** 10.1007/s00127-022-02361-4

**Published:** 2022-09-16

**Authors:** Amelia Ishikawa, Debra Rickwood, Emily Bariola, Navjot Bhullar

**Affiliations:** 1 Faculty of Health, University of Canberra, Canberra, ACT Australia; 2 Kantar Public, Melbourne, VIC Australia; 3 Discipline of Psychology, Edith Cowan University, Perth, WA Australia

**Keywords:** Self-reliance, Help-seeking, Perceived social support, Resilience, Youth, Mental health problems

## Abstract

**Purpose:**

Many young people with mental ill-health do not seek support, and developmental growth in self-reliance may be a barrier to help-seeking. Increasing autonomy is a positive developmental task for youth and a key aspect of resilience. This study examined the influence of perceived social support and resilience on the previously unexamined relationship between self-reliance and intentions to seek help from informal, professional, and self-help sources for mental health problems.

**Methods:**

An online survey was completed by a representative Australian community sample of 5,203 young people aged 12–25 years (half female), in May–June 2020.

**Results:**

Path analysis showed the hypothesised conceptual model did not fit the data well, but a modified model was a good fit. Higher self-reliance was associated with lower intentions to seek informal and professional help, as expected, but not with greater intentions for self-help. The relationship between self-reliance and informal help-seeking intentions was fully mediated by perceived social support, whereas the relationship between self-reliance and professional help-seeking was also direct. Perceived social support fully mediated the relationship between self-reliance and resilience. Intentions to use self-help were not influenced by variables in the study, but higher self-help intentions were associated with higher professional help-seeking intentions. Associations were consistent across age and gender groups.

**Conclusion:**

The results show the critical role of social support for combating some of the unhelpful aspects of self-reliance for mental health help-seeking in young people. Future research should explore how self-reliance can hinder or be harnessed to facilitate accessing appropriate mental health.

**Supplementary Information:**

The online version contains supplementary material available at 10.1007/s00127-022-02361-4.

## Introduction

Young people are in a dynamic period of change and development characterised by increasing levels of autonomy and personal agency [1, 2]. Paradoxically, it is during the adolescent and early adult years (approximately 12–25 years) when young people are shifting away from parental dependence, that there is increased vulnerability to mental illness requiring treatment and support from others. Early adolescence is the most likely age of onset for mental health disorders with half of lifetime disorders beginning before the mid-teen years [3]. In a national survey of 2,967 Australians, 7.7% of 11–17-year-olds self-reported depression and one in 10 indicated they had engaged in self-harm [4]. Despite high levels of need, young people (16–24 years) are the least likely group to engage with professional help for mental health issues [5, 6]. By not engaging with services in a timely manner, mental health problems may increase in severity and have ongoing detrimental impacts as they persist into adulthood [7, 8]. Consequently, the barriers to seeking help among young people must be well understood and mitigated to ensure young people receive the mental health support they need.

### Help-seeking for mental health problems

Help-seeking occurs when a symptomatic individual identifies a need and takes steps to meet that need [9]. Rickwood et al. [10] propose the help-seeking process as a multi-staged, non-linear model, with decision points at each stage. Decisions made throughout the process impact on whether the individual chooses to proceed with their intention to seek help, the type of help accessed, and the extent of their engagement with the identified source of support. Rickwood’s help-seeking process model [11] proposes that help-seeking commences with the onset of symptoms followed by the individual recognising that they need help to manage those symptoms. Once the need for help is determined, individuals must decide on what type of help they require and gauge their willingness and readiness to engage. The process moves beyond intentions and into behaviour when the individual approaches the selected source of help and engages, with the goal of reducing their distress.

Help-seeking is categorised according to the source of support being sought, and includes formal, semi-formal, informal, and self-help sources [11]. Formal support describes help sought from professionals trained to provide relevant services, such as psychologists or general practitioners. Semi-formal support is provided by non-mental health professionals, such as teachers [10, 11]. Informal support is offered by friends, intimate partners or family; people who are connected to the individual via social ties [5]. Young people prefer to access informal support, particularly from family, friends, and partners [10]. Finally, self-help describes support an individual seeks independently, such as accessing resources and information online [12].

### Barriers and facilitators of help-seeking

Barriers to help-seeking are better understood than facilitators, as most research has focussed on identifying obstacles that prevent people from reaching out for help. Individual-level factors, such as a preference for self-reliance and the lack of ability to identify symptoms, are key barriers to help-seeking for young people [13, 14]. Social factors such as shame, perceptions of stigma, or concerns about confidentiality also often prevent young people from seeking help, as do structural barriers including cost, wait times, and limited access to services [13, 14]. The key facilitators of help-seeking are encouragement from social supports, prior positive experiences of help-seeking, and trust in the confidentiality of services [13, 14].

### Self-reliance

Unsurprisingly, a key barrier to help-seeking for young people is the preference to manage problems on their own, as this period of life is characterised by a normative positive increase in autonomy, initiative, and control [15, 16]. Despite being an important milestone, the developmental shift towards independence can create challenges for young people struggling to balance their competing needs for autonomy and support [17, 18]. During adolescence, young people shift away from dependence on their parents, and this is usually coupled with increasing reliance on peers for advice and support [16, 19]. This suggests adaptive adolescent autonomy is marked by a shift in who young people turn to for support, rather than a disconnection from all support networks. Developing autonomy is characterised by an increase in independent thinking and decision-making that flourishes within the context of healthy relationships [20]. Being unable to accommodate appropriate interdependence is a maladaptive expression of this important developmental task [21] that can see adolescents attempting to self-manage problems beyond their capacity. For example, Labouliere et al. [22] found that severely depressed young people who endorsed attitudes of extreme self-reliance–the preference to manage problems on their own all of the time–were less likely to draw on informal support and preferred to seek help independently from online sources.

More extreme manifestations of self-reliance have been associated with poor evaluations of available social support [23, 24]. Although a broad concept, social support is defined as receiving, or perceiving availability of, support provided by one’s social network, particularly to help cope with stress [25, 26]. Beyond its contribution to stress and coping, social support is fundamental to human survival and is associated with physical and psychological wellbeing and flourishing [27]. Social support can take multiple forms such as emotional (comfort), informational (advice), and instrumental (practical help) [28]. It can be measured in terms of both actual received support and perceived support. Perceived support is how one judges the availability of support and is influenced by subjective appraisals, judgements [29], individual traits, and attachment style of the recipient [24, 30]. Perceived support has been shown to be more important than actual support [31]. Notably, perceived social support has a significant impact on whether individuals seek out their network in times of need [25].

Some young people, such as those who have experienced unstable family environments, are particularly vulnerable to experiencing extreme levels of self-reliance and low levels of help-seeking [32, 33]. The experience of consistently unresponsive care during infancy has been associated with the tendency to evaluate others, and their capacity to provide beneficial support, negatively. These negative evaluations can lead to excessive self-reliance and reduced intentions to seek support [24]. In such situations, extreme self-reliance is a self-preserving response to perceptions of inadequate social support rather than an adaptive shift towards autonomy. Autonomy without connection has been associated with a reduced capacity to draw on help when required [15].

The relationship between self-reliance and resilience, which is the ability to bounce back following adversity [34], is complex and largely linked to social influences. A positive social environment can nurture a sense of personal agency and a healthy balance between self-reliance and connectedness [21, 25, 35]. However, a dysfunctional social environment can cultivate rigid independence and resistance to any support [33]. Although seemingly maladaptive, for young people facing adversity, self-reliance may be an expression of resilience, as they are demonstrating the capacity to adapt and cope in difficult circumstances. However, high levels of self-reliance are associated with a rigid, self-imposed expectation that the individual should be able to manage problems independently [36, 37]. Other facets of resilience, such as flexibility and connectedness, do not align with the unwavering independence observed in individuals with high levels of self-reliance [21, 38]. Self-reliance may be viewed as an aspect of resilience, conceptualised as a belief in one’s own ability to draw on personal capacities and past successes in the navigation of difficulties [39]. However, while self-reliance can be an effective strategy to cope with minor challenges, it may prevent individuals from seeking as well as accessing appropriate supports when required [40]. Ungar [41] identifies the balance between independence and the ability to draw on meaningful support when needed as an expression of resilience. Understanding more about the interplay between self-reliance, perceived social support, and resilience could provide useful insights into how to ensure that the natural drive for adolescent autonomy still allows for adaptive help-seeking.

### The present study

**Fig. 1:**
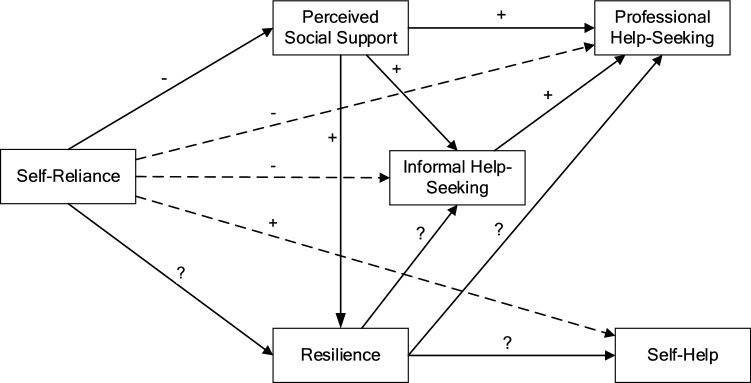
Hypothesised relationships between self-reliance, perceived social support, resilience, professional help-seeking, informal help-seeking, and self-help. Note. Dashed lines represent relationships hypothesised as mediated or partially mediated; plus sign (+) = hypothesised positive association; minus sign (− ) = hypothesised negative association; question mark (?) = exploratory investigation

This study extends previous research identifying self-reliance as a barrier to help-seeking by exploring the relationship between the preference for self-reliance and intentions to seek help from various sources, and how these relationships are influenced by perceived social support and resilience. Figure 1 summarises the hypothesised relationships. Specifically, we predicted that:

1. Individuals with higher self-reliance would report lower levels of informal and professional help-seeking and higher levels of intentions to seek self-help,
2. Individuals with higher levels of self-reliance would report lower levels of perceived social support,
3. The relationship between self-reliance and intentions to seek informal and professional help would be mediated by perceived social support, and
4. Individuals with higher intentions to seek informal help would report higher intentions to seek professional help.
5. We also explored the relationship between self-reliance and resilience and whether resilience mediated the relationship between self-reliance and intentions to seek help from different sources (informal, professional, self-help). As these were exploratory analyses, no specific a priori hypotheses were proposed.
6. Finally, due to the major developmental changes that take place between the ages of 12 and 25 years, we examined whether the hypothesised pathways were invariant across age-groups and gender. We anticipated that, although there were likely to be age and gender differences in the mean levels of help-seeking, self-reliance and social support, the interrelationships among these measures would not vary across age and gender.

## Methods

### Participants

The participants comprised a nationally representative sample of 5,203 Australian young people aged 12–25 years. There were 74% (*n* = 3,843) of participants residing in metropolitan locations and 26% (*n* = 1,359) from regional areas. Recruitment ensured an even spread of participants across four age groupings: 12–14 (*n* = 1,282), 15–17 (*n* = 1,286), 18–21 (*n* = 1,305), and 22–25 (*n* = 1,330) years. Males and females were equally represented, with 2,584 males (50%) and 2,575 females (50%); 1% (*n* = 37) of the participants indicated ‘other’ as their gender. There were 6% who were Aboriginal or Torres Strait Islander (*n* = 307).

## Measures

Participants responded to a 93-question online self-report survey measuring a number of mental health-related constructs. Along with key demographic questions, the following measures were used in the current study.

### Help-seeking intentions

This study used an adapted version of the General Help-Seeking Questionnaire (GHSQ) [42] that measures participants’ intentions to seek help by indicating how likely they were to draw on each source of support using a Likert-type scale (1 = extremely unlikely; 7 = extremely likely)*.* The various sources of help were divided into three subscales: informal help (mother, father, family, friend, partner) (*α*=0.62), professional help (GP, mental health professional) (*α*=0.72), and self-help (internet) for analysis. A composite score was calculated for each of the subscales by averaging overall scores. Scores ranged between 1 and 7 and higher scores indicated higher intentions to seek support from that source. The GHSQ has demonstrated strong internal consistency in studies involving young people [43, 44] and in the current study (α=0.73)

### Perceived social support

Perceived social support was measured using the Multidimensional Scale of Perceived Social Support (MSPSS) developed by Zimet et al. [45]. The dimensions of support measured include the overall appraisal of social support adequacy and the subjective perception of support available from multiple sources (significant others, family, friends). It is a 12-item measure, answered on a 7-point Likert-type scale whereby 1 = very strong disagreement and 7 = very strong agreement. Questions include “There is a special person who is around when I am in need,” and “My family really tries to help me.” The total score is an average of responses on all items, ranging from 1 to 7, and higher scores indicating higher perceived levels of social support. In this study, the measure demonstrated strong internal reliability (*α*=0.93).

### Resilience

Resilience was measured using the Brief Resilience Scale (BRS) [34] that specifically measures one’s ability to bounce back from stressful situations. The measure comprises six questions, alternating between positive and negative wording (reverse scored). Questions are answered on a 5-point Likert-type scale whereby 1 = strongly disagree and 5 = strongly agree. A composite score was created by averaging all scale items, with higher scores indicating greater levels of resilience. The BRS demonstrated good internal reliability in the present study (*α*=0.81).

### Self-reliance

A measure of self-reliance was developed specifically for this study. Three relevant items were drawn from different sections of the survey. Individuals who indicated they were more likely (score of 5 or above) to seek help from no one (drawn from the GHSQ) were identified as endorsing a preference for self-reliance. The second item was drawn from a list of survey items used to measure barriers to help-seeking [46]. The item asked “If you were experiencing a personal or emotional problem, would you speak to someone or try to deal with the problem by yourself?” Those who selected “deal with it on my own” were identified as having a preference for self-reliance. Individuals who subsequently selected their reason as “I prefer to sort out emotional/personal problems on my own” were classified as having a preference for self-reliance. A composite score was created by averaging overall scores, ranging between 0 and 3, with higher scores indicated higher preference for self-reliance. These three items demonstrated adequate internal consistency with *α *= 0.62. There is no current scale designed to specifically measure a preference for self-reliance in young people regarding mental health issues. Previous studies have similarly used only one or two items to measure self-reliance [22, 40].

## Procedure

This cross-sectional study used data from the National Youth Mental Health Survey commissioned by headspace (Australia’s National Youth Mental Health Foundation) and conducted by Kantar Public (a public sector research and evaluation consultancy). Ethical approval was provided by Belberry Limited Human Research Ethics Committee (reference: 2020–04-395).

Participants were recruited via an online panel comprising more than 400,000 Australians over the age of 18 who have signed up to participate in surveys. Respondents were screened to ensure the sample was representative across age, gender and geographic location. Participants aged 12–17 years were recruited through their parents/guardians who were part of the panel. Parents/guardians provided consent for those under 18 years, and participants provided consent before participation. Data were de-identified and could not be linked back to individual responses. The survey took approximately 25 min to complete. Data were collected between 25 May and 21 June 2020.

## Results

Significance was set at *p* < 0.001 to eliminate trivial effect sizes due to the very high power from such a large sample size. Descriptive statistics are presented for the key study variables in Table 1. The mean score for resilience was just above the scale mid-point, indicating a central distribution. Perceived social support had a mean score 1.3 points above the scale mid-point, indicating participants had high perceived social support. The self-reliance measure showed considerable variance, with the standard deviation being slightly larger than the mean. For the help-seeking measures, intentions to seek informal help recorded a notably high mean and large skew, with participants on average indicating they were highly or extremely likely to draw on informal sources. Mean scores for professional help-seeking and self-help were close to the scale mid-point.

**Table 1:** Descriptive statistics for all predictor and outcome variables

Variable	*N*	Possible range	*M*	SD	Skewness (SE)	Kurtosis (SE)
Resilience	5,203	1–5	3.25	0.69	0.00 (0.03)	0.12 (0.07)
Perceived social support	5,203	1–7	5.30	1.00	− 0.74 (0.03)	0.74 (0.07)
Self-reliance	5,203	0–3	0.85	1.00	0.79 (0.03)	− 0.66 (.07)
Informal help-seeking	5,198	1–7	6.56	0.82	− 2.55 (0.03)	8.83 (0.07)
Professional help-seeking	5,030	1–7	4.48	1.76	− 0.39 (0.04)	− 0.75 (0.07)
Self-help	5,040	1–7	4.21	1.84	− 0.27 (0.03)	− 0.93 (0.07)

Analysis of variance revealed that there were significant age-group and gender effects for most variables; the exception being professional help-seeking where there were no demographic differences (see Table S1). Briefly, the adolescents aged 12–14 and 15–17 years had higher resilience, higher social support, lower self-reliance, and higher informal help-seeking than the young adults aged 18–21 and 22–25 years. The only gender effect was evident for resilience, where males had higher levels of resilience than females. For self-help, there was a significant interaction, such that there was no age effect for females, but a significant linear age effect for males, with increasing self-help with older age.

Most hypothesised bivariate relationships demonstrated significant, small to moderate associations (see Table 2). Higher levels of self-reliance were weakly associated with lower intentions to seek all three sources of help. Individuals with higher self-reliance had lower perceived social support and lower resilience. Resilience and social support were moderately associated. The help-seeking measures were all weakly interrelated.

**Table 2:** Intercorrelations

Variable	1	2	3	4	5	6	7
1. Gender (male)	–						
2. Age	− 0.01	–					
3. Social support	− 0.03	− 0.22^*^	–				
4. Resilience	0.09^*^	− 0.19^*^	0.40^*^	–			
5. Self-reliance	0.02	0.21^*^	− 0.37^*^	− 0.17^*^	–		
6. Informal help	− 0.05	− 0.09^*^	0.53^*^	0.20^**^	− 0.23^*^	–	
7. Professional help	.03	-.01	.18^*^	-.03	-.19^**^	.20^*^	-
8. Self-help	.04	.07^*^	-.01	-.06^*^	-.08^*^	.06^*^	.24^*^

*Note. N* = 4992–5040 due to missing data. ^*^p < .001

Higher perceived social support was moderately associated with higher intentions to seek help from informal sources; this was the strongest relationship. There was a weak positive relationship between social support and intentions to seek professional help, but no significant relationship between social support and self-help. Higher levels of resilience were weakly associated with higher intentions to seek informal help and very weakly related to lower intentions to seek self-help. For the demographics, being male was weakly associated with higher resilience, and older age was weakly associated with lower social support, lower resilience, and lower informal help-seeking, but slightly higher intentions to seek self-help.

### Path model predicting help-seeking intentions

A path analysis using structural equation modelling was conducted to test the hypothesised path model outlined in Fig. 1. The model fit indices and criteria used included goodness of fit index (GFI) = 0.95, adjusted goodness of fit index (AGFI) = 0.95 [47], Tucker Lewis index (TLI) close to 0.95 [48], and the root mean error of approximation (RMSEA) ≤ 0.07 [49]. The criteria for adequate fit using *χ*^2^ is a non-significant *p* value [50], however, due to the impact of large sample size on chi-squared test value, RMSEA was chosen as the key index to determine model fit [51]. Table 3 presents a summary of the model fit indices, revealing that the hypothesised model was not a good fit. By removing non-significant paths and including a path from self-help to professional help-seeking, a good-fitting model was obtained.

**Table 3:** Summary of model fit indices

Model	*N*	*Χ*^2^	df	*p*	GFI	AGFI	TLI	RMSEA
Hypothesised model	5203	368.068	3	< .001	.978	0.844	0.568	0.153
Modified model	5203							
Unconstrained^a^		223.066	56	< 0.001	.986	0.959	0.909	0.024
Fully constrained^b^		398.476	112	< 0.001	0.975	0.963	0.922	0.022
Difference^ab^		175.410	56	< .001				
Partly constrained^c^		307.619	105	< 0.001	0.981	0.969	0.941	0.019
Difference^ac^		84.553	49	> 0.001				

Unconstrained^a^ all regression paths able to vary by age-group and gender; Fully constrained^b^ all regression paths invariant across age-group and gender; Partly constrained^c^ all regression paths invariant across age-group and gender, except path from social support to resilience which is unconstrained and able to vary across age-group and gender

**Fig. 2:**
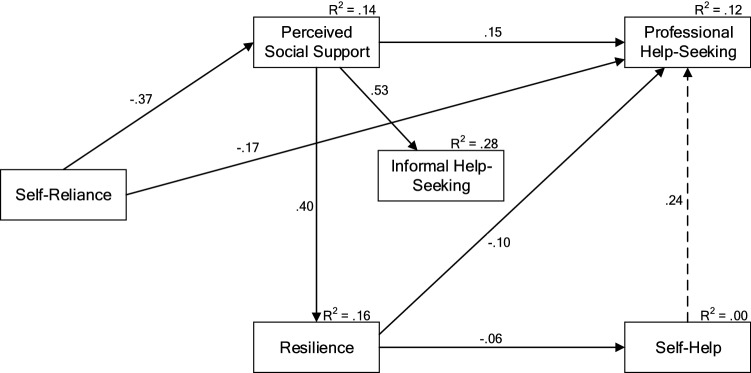
Final modified model demonstrating relationships between self-reliance, perceived social support, resilience, professional help-seeking, informal help-seeking, and Self-Help. Note. All paths are significant at *p* < 0.001. The post-hoc modified path is represented by a dashed line. All paths are invariant across age and gender group, except for the path from perceived social support to resilience (0.40), which ranged from 0.25 to 0.45 across age by gender groups

The modified path model (Fig. 2) showed perceived social support fully mediated the relationship between self-reliance and intentions to seek informal help. Higher levels of self-reliance were associated with less perceived social support, which in turn was associated with decreased intentions to seek informal help (positive relationship between perceived social support and informal help-seeking). Perceived social support similarly fully mediated the relationship between self-reliance and resilience, such that higher levels of self-reliance were associated with less perceived social support, which in turn was associated with lower levels of resilience (positive relationship between perceived social support and resilience).

The model confirmed a direct relationship between self-reliance and intentions to seek professional help, whereby higher self-reliance was associated with decreased intentions to seek help professionally. This relationship was also partially mediated by perceived social support. Higher levels of resilience directly, but weakly, predicted lower self-help and professional help-seeking intentions. The model also indicated a direct effect between intentions to use self-help and professional help, which was not hypothesised. The squared multiple correlations showed 28% of variance in intentions to seek informal help and 12% variance in professional help-seeking intentions were explained. The model also explained 14% variance in perceived social support and 16% variance in resilience, but accounted for negligible variance in intentions to use self-help.

This modified model was unconstrained by age-group and gender, and showed a good fit for all the data combined as well as for each of the eight data groups (age-group by gender). To determine whether the hypothesised pathways were invariant across age-groups and gender, a fully constrained model was then fit, imposing invariance across age by gender groups on the regression coefficients for each pathway in the model. This fully constrained model was significantly different from the unconstrained model, meaning that the model was not invariant across age and gender groups. Examination of differences in regression coefficients revealed that the pathway from social support to resilience may not be invariant. Consequently, the model was rerun allowing this pathway to be unconstrained, which resulted in a model that was not significantly different from the fully unconstrained model, and a good fit to the data. The regression coefficients for this pathway showed that although the effect was in the same direction for each age by gender group, the strength of the effect was greater for the adolescent age groups and was weakest for the oldest males (standardised regression coefficients—Females: 12–14 years = 0.44, 15–17 years = 0.44, 18–21 years = 0.34, 22–25 years = 0.33; Males: 12–14 years = 0.40, 15–17 years = 0.45, 18–21 years = 0.36, 22–25 years = 0.25).

## Discussion

The aim of this study was to examine the relationship between self-reliance and intentions to seek help from informal, professional, and self-help sources, and to understand the roles of perceived social support and resilience in this relationship. As predicted, the multivariate model results demonstrated that higher self-reliance was associated with lower help-seeking from informal sources, although this relationship was fully mediated by perceived social support, with higher self-reliance associated with lower levels of perceived social support, which in turn was associated with lower informal help seeking intentions. However, higher self-reliance retained a direct influence on help-seeking intentions from professional sources, and this was not fully mediated by perceived social support as predicted. Higher perceived social support was associated with higher levels of resilience, as anticipated, but also fully mediated the relationship between self-reliance and resilience, which was not anticipated. Our multivariate results showed no significant association between intentions to seek informal help and professional help-seeking, as was expected [52], but rather a significant positive relationship between self-help and professional help-seeking intentions.

In concert with previous research [14, 17], the bivariate correlations in our results also showed that individuals with a preference for self-reliance were less likely to intend to seek help from professional or informal sources. Previous studies have found similarly small but significant correlations between the need for autonomy and reduced intentions to seek help from informal support or mental health services [53]. The relationship between self-reliance and informal help was fully mediated by the relationship between self-reliance and social support and this was partly the case for professional help-seeking, showing the impact of perceived social support.

Young people with higher self-reliance were slightly more likely to intend to use self-help via the internet, as predicted. The main predictor of online help-seeking has been shown to be high levels of psychological distress [54, 55], which we did not control for in this study. The online environment can provide immediate and generally anonymous support, where young people can retain control over the help-seeking interaction, which would be valued by young people with higher levels of self-reliance.

Our results add quantitative evidence for a relationship between higher self-reliance and less perceived social support, as identified in prior qualitative research [32, 33]. High self-reliance appears to act as a barrier to accessing mental health supports through less social support. Perceived social support has been previously found to mediate informal help-seeking intentions in young survivors of dating violence [56], and the help-negating effect in American college students [44].

We found a direct relationship between a higher preference for self-reliance and lower intentions to seek professional help. Wilson et al. [53] also observed a direct relationship in young people expressing a need for autonomy and reduced help-seeking from professional sources. This direct relationship could be explained by factors such as self-stigma [22], lack of trust in services, belief that nothing can help [57], and fear of embarrassment [57]. The attitudinal underpinnings of self-reliance may play a role in inhibiting professional help-seeking beyond the influence of perceived social support. The current study did not measure other attitudinal barriers, so these links remain speculative.

As predicted, there was a strong positive relationship between perceived social support and resilience. This is well-supported by previous studies [25, 58], but very little prior research has examined the relationship between self-reliance, resilience, and help-seeking. This study adds that resilience does not influence the relationship between self-reliance and help-seeking, except that the relationship between self-reliance and resilience was fully mediated by perceived social support, highlighting the considerable influence of support networks on help-seeking and wellbeing processes.

Notably, the BRS used to measure resilience in this study specifically measures the sense of self-agency to recover from stress [59]. The lack of a direct relationship between self-reliance and resilience suggests that the belief that one can easily bounce back following adversity is not necessarily contingent on self-reliance. Rather, perceptions of available social support seem to have more influence on how well a young person thinks they can recover. Interestingly, resilience had weak but significant negative relationships with self-help and professional help-seeking, whereby higher resilience predicted lower intentions to draw on both sources of help. Previous research by Schomerus et al. [60] found a similarly weak relationship between higher resilience and lower intentions to seek professional help. The chosen resilience measure may contribute to these findings: those with a high sense of agency to recover from stress may express lower intentions to seek help as they might feel confident of their capacity to recover on their own or with help from informal networks.

### Strengths and implications

This study benefitted from a large, representative sample of young people, which enables the results to be generalisable within an Australian context. Although the effects found are small, the findings increase knowledge of factors influencing the relationship between self-reliance and help-seeking. We highlight that young people with higher self-reliance tend to perceive lower levels of social support, and that greater social support is important for seeking help from informal and professional sources. There is strong consensus in the literature that informal supports are both preferred by young people and key facilitators into professional services [13, 52], so this impact of self-reliance can be maladaptive. Self-reliance also retains a direct impact on lower intentions to seek professional help.

Understanding the cascading relationships between stronger preferences for self-reliance, more negative appraisals of available support, and reduced intentions to seek help from informal networks, can inform early interventions aimed at increasing service use in young people. The first implication of our findings is that to encourage early intervention for young people with high self-reliance (and associated lower perceived access to social support that can, in turn, hinder help-seeking), special attention needs to be paid to reaching out to these young people and framing help-seeking in a way that defines it as a resource to support their independence, rather than a reliance on others for help. The second implication, relevant to service providers, is the need to recognise the potential for self-reliance to be maladaptive. There is an important balance between promoting healthy independence, while identifying and mitigating maladaptive levels of self-reliance that reduce active coping through informal help-seeking. Service providers should ask about young people’s views on the importance of self-reliance for them, and discuss how this may affect their resilience through their social support, and how it may affect future help-seeking. Our results suggest that the relationship between self-reliance and resilience via perceived social support has important implications for physical and mental health outcomes for young people because of its impact on help-seeking [61, 62].

Perceived support is largely determined by trait influences (biological predispositions, learned tendencies, experiences of receiving support, attachment styles) and social influences (actual or imagined interactions with support) [30, 31]. Although a relatively stable construct [63], previous research has found psychoeducational interventions focussed on improving self-esteem and self-concept to be effective in improving perceptions of support [64]. Nurturing perceptions of social support within a holistic framework, particularly for young people identified as being highly self-reliant, may decrease the risk of disconnection while also increasing the young person’s sense of self-efficacy for coping with adversity.

### Limitations and recommendations

Important methodological limitations include that the measure of self-reliance was not a standardised or validated measure; rather, it was compiled by extracting items relating to self-reliance from other measures. Other studies of self-reliance and help-seeking have used similar measures containing only one or two items [22, 65]. Currently there is no measure of preference for self-reliance in the management of mental health problems for young people. Available self-reliance measures have been designed for specific populations (e.g., adult males) [66], or within particular contexts such as work settings or personal relationships [67]. Future research should develop a measure of self-reliance specific to managing mental health and wellbeing. This would support further research into how high levels of self-reliance impact help-seeking, and how this can be addressed to ensure that young people better access services when required. In the context of mental health care (and possibly all health care), there is an optimal balance between autonomy and support seeking, with young people needing to overcome their tendency toward self-reliance when their health needs professional attention,

The results of our study come from cross-sectional data, meaning the relationships cannot be interpreted as causative or sequential. Further, we investigated help-seeking intentions, which, although predictive of help-seeking behaviours [42], are not equivalent and other factors impact actual behaviour. For example, we did not consider the impact of mental health status on help-seeking outcomes. Future longitudinal studies should also consider levels of distress, which have been found to influence help-seeking intentions and levels of self-reliance [17, 22].

## Conclusion

This research adds to knowledge relating to the influence of self-reliance on intentions to seek help from key sources. How young people appraise the availability and reliability of their support networks strongly influences whether they intend to turn to informal supports for help and affects professional help-seeking. Social support also impacts on their sense of personal agency to deal with adversity. This study reinforces the importance of prioritising initiatives aimed at improving perceived social support to help young people successfully navigate their mental health needs during a time of growing independence. Although control and decision-making capacity develop with maturity, young people continue to need the support of family and, increasingly, peers as they develop in autonomy. Finding ways to increase positive appraisal of social support for young people who value self-reliance may be integral to promoting a healthy balance between independence and the ability to seek support when needed. If young people can draw on their informal networks for support, they are more likely to engage help if their needs escalate. Improving their uptake of self-help resources could also be a good place to intervene to improve options for self-reliant young people to reach out when in need.

## Supplementary Information

Below is the link to the electronic supplementary material.

Supplementary file1 (DOCX 14 KB)
