# Supplementary material for: Autonomy versus support: self-reliance and help-seeking for mental health problems in young people
Source: Soc Psychiatry Psychiatr Epidemiol. 2022 Sep 16;58(3):489–99. doi: 10.1007/s00127-022-02361-4 (PMC9971084; doi:10.1007/s00127-022-02361-4)
Supplement: Supplementary file 1 — Supplementary file1 (DOCX 14 KB) [file 127_2022_2361_MOESM1_ESM.docx]

Table S1. Mean scores by gender and age-group

|  |  |  |  | Variable |  |  |  |
| --- | --- | --- | --- | --- | --- | --- | --- |
| Age Group | N | Resilience | Social support | Self-reliance | Informal help-seeking | Professional help-seeking | Self-help |
| Male | | | | | | | |
| 12-14 | 652 | 3.44 | 5.59 | 0.60 | 6.67 | 4.43 | 4.00 |
| 15-17 | 644 | 3.47 | 5.58 | 0.63 | 6.60 | 4.54 | 4.16 |
| 18-21 | 640 | 3.21 | 5.06 | 1.11 | 6.41 | 4.50 | 4.41 |
| 22-25 | 648 | 3.31 | 5.05 | 1.12 | 6.53 | 4.58 | 4.52 |
| Female | | | | | | | |
| 12-14 | 623 | 3.36 | 5.71 | 0.55 | 6.66 | 4.51 | 4.16 |
| 15-17 | 624 | 3.34 | 5.60 | 0.60 | 6.65 | 4.57 | 4.10 |
| 18-21 | 656 | 3.03 | 5.04 | 1.12 | 6.51 | 4.14 | 4.06 |
| 22-25 | 672 | 3.12 | 5.18 | 1.03 | 6.56 | 4.45 | 4.22 |
